# Application of three-dimensional printing technology in peripheral hip diseases

**DOI:** 10.1080/21655979.2021.1967063

**Published:** 2021-09-03

**Authors:** Shuai Liang, Jia Xie, Fangyuan Wang, Juehua Jing, Jun Li

**Affiliations:** Department of Orthopedics, The Second Hospital of Anhui Medical University, Hefei, Anhui, China

**Keywords:** Tree-dimensional (3d) printing, developmental dysplasia of the hip (ddh), periacetabular tumors, acetabular fracture, periprosthetic hip joint infection (pji)

## Abstract

The incidence of peripheral hip diseases is increasing every year, and its treatment is always tricky due to the complexity of hip joint anatomy and a variety of surgical methods. This paper summarizes the application research and progress of three-dimensional (3D) printing technology in different peripheral hip diseases in recent years published by PubMed from January 2017 to July 2021 with the search terms including “3D or three-dimensional, print*, and hip*. In general, the application of 3D printing technology is mainly to print bone models of patients, make surgical plans, and simulate pre-operation, customized surgical navigation templates for precise positioning or targeted resection of tissue or bone, and customized patient-specific instruments (PSI) fully conforms to the patient’s anatomical morphology. It mainly reduces operative time, intraoperative blood loss, and improves joint function. Consequently, 3D printing technology can be customized according to the patient’s disease condition, which provides a new option for treating complex hip diseases and has excellent application and development potential.

## Introduction

Peripheral hip diseases are common orthopedic diseases, including DDH, acetabular fracture, periacetabular tumors, PJI. It has been reported that their global incidence increases with population growth and with age, year by year [1–3], especially in China. To date, the most common clinical treatment method for peripheral hip diseases is surgery. However, the surgery is challenging because of the complex deep anatomy of the hip and the narrow safe passage required. Moreover, this approach relies heavily on the operator’s clinical experience for the recognition of anatomical sites and lesion sites.

Three-dimensional printing technology, also known as rapid prototyping (RP), is based on imaging data, on the strength of the principle of layered manufacturing. It uses laser or electron beams to stack 2D cross-sectional shapes on top of cohesive materials such as thermoplastic or liquid metals to create 3D scale models of physical objects quickly [4]. Clinical applications of it in orthopedic departments mainly include 3D printing of a bone model, custom prostheses, and navigation templates. It can significantly help orthopedic surgeons to plan surgery and provide patients with the best treatment plan for the disorder.

Medical applications of 3D printing date back to the early 2000s [5], such as, 3D printing fabricate scaffolds based on Hydroxyapatite [6], 3D organ printing technology combining an engineering approach with the developmental biology concept of embryonic tissue fluidity [7]. More recently, along with the development of 3D printing technology in orthopedic surgery, it has achieved positive results, providing new treatment solutions and ideas for treating hip diseases. For example, the investigation of osseointegration in recovered 3D-printed acetabular implants Greater bone ingrowth was seen in 3D-printed implants, suggesting that improved osseointegration is possible [8].

In this study, we screened and reviewed articles that were guided by a PubMed search of original and review articles on the application of 3D printing in a spectrum of peripheral hip diseases from January 1,2017 to July 31,2021. The search terms including “3D or three-dimensional, print*, and hip*. At the same time, this study selected clinical research literature that included clinical control groups, a large sample size, and more mid-term and long-term follow-up. We believe that 3D printing technology has promising results and future prospects in hip diseases, and we hope that by summarizing and exploring current relevant papers in this paper, we will be able to better understand the effects of 3D printing technology, as well as its latest developments and future research breakthroughs.

## Developmental dysplasia of the hip

DDH refers to a variety of abnormal hip development conditions, ranging from mild acetabular dysplasia without hip dislocation to complete hip dislocation during growth and development [9]. Acetabular dysplasia is usually characterized by a shallow or vertically oriented acetabulum with inadequate femoral head coverage, thereby contributing to irreversible aggravation as the joint matures, resulting in persistent claudication, pain, osteoarthritis, and affects the patient’s daily life [10,11]. Accordingly, most patients eventually undergo total hip arthroplasty (THA) to provide long-term pain relief while improving hip function. However, anatomical variations in hip dysplasia leads to difficult and risky surgical operations. To date, the surgeon used the imaging results as the basis of the surgical plan, combined with personal experience and intraoperative specifics [12]. However, the application of 3D printing technology has unique advantages, showing a great specifically therapeutic effect in clinical trials for DDH therapy. This concept’s anatomical correction and biomechanical stability were tested in a canine model that, like humans, suffers from hip dysplasia, demonstrating that patient-specific shelf implants significantly improved the coverage and stability of dysplastic hips in a canine model with naturally occurring hip dysplasia [13].

The individualized 3D printed 1:1 pelvis model can help the surgeon recognize the anatomical variation of the acetabulum and the degree of damage, which allows the surgeon to develop a reasonable surgical plan and perform preoperative simulation [14], to improve the success rate of surgery [15], guide the selection of prosthesis, reduce the intraoperative selection time, and improve the accuracy of placement. Recently, a successful operation was performed on an old man, assisted by the model. Preoperative planning was based on a 3D model for accurate anatomical assessment and preoperative training. The results showed that the model improved the diagnostic accuracy and helped determine the implant and implant size in advance [14].

**Figure 1.**
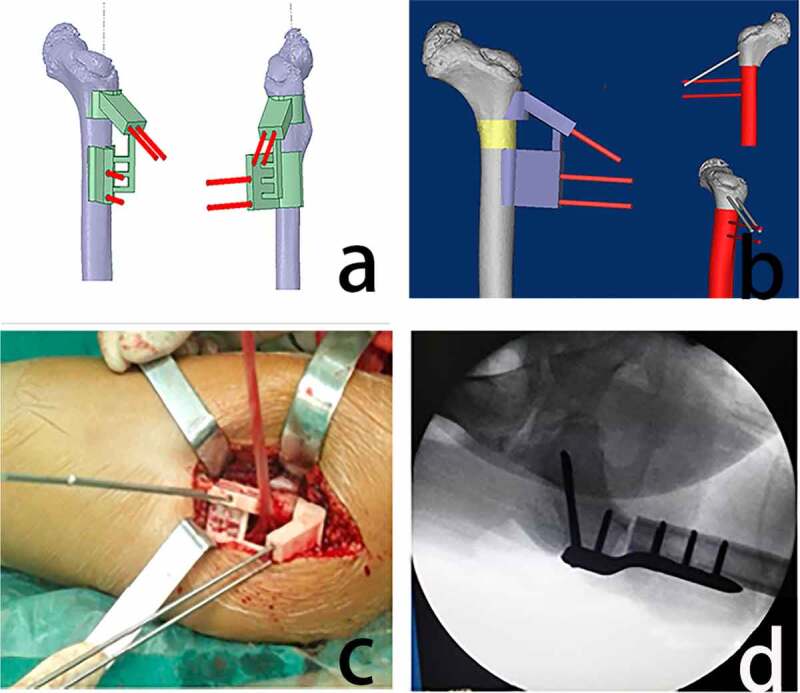
Design and use of the 3D printed navigation template (a) the 3D navigation template model and the kirschner wire channel were reverse designed according to the 3D reconstruction of the femur. (b) A navigation template was used to simulate the osteotomy process using the kirschner wire as a lever. (c) the navigation template was accurately positioned at the femoral osteotomy during surgery. (d) the intraoperative examination was performed using C-arm x-rays [19]

Furthermore, 3D printing technology has also been used to make surgical navigation templates, which can be accurately positioned [16], effectively reducing errors caused by inexperience [17], reducing intraoperative operation time and blood loss, and simplifying the surgical process [18,19], It has also been proposed that 3D printed guides can increase the precision of femoral anteversion repair during hip arthroplasty [20]. Zheng et al. [19]compared a 3D-printed navigation template for proximal femoral osteotomy in older children with hip dysplasia to 13 patients who underwent the same procedure but did not have a navigation template. As a result, they found that the operation time, the number of X-ray exposures and the occurrence of femoral epiphysis damage were significantly decreased (P < 0.05) (Figure 1). Ferretti discovered that using laser-guided implantation of patient-specific devices in total hip arthroplasty also improved placement accuracy substantially [21].

Patients with acetabular deformities have individual differences, and traditional prostheses can’t meet the needs of all patients, 3D printing individual customized prostheses to solve this issue [22]. Geng [23]followed 92 patients who underwent THA using 3D-printed porous trabecular titanium acetabular cups for an average of 48.2 months and showed that the acetabular cups were precisely matched and stable. Similarly, Qiang et al. [24]performed femoral osteotomy and acetabular reconstruction in 12 patients using a 3D printing prosthesis. Follow-up results showed significant improvement in the Harris hip score and affected limb deformity. There is no doubt that the performance of the 3D printed prosthesis is directly related to the success of THA surgery and the patient’s quality of life [25]; nevertheless, Hothi [26] found that the 3D printed acetabular cups have structural cavities. In contrast, conventional cups have no discernible holes at all, which may affect their mechanical properties.

## Periacetabular tumors

The hip is the most common location of primary and metastatic bone tumors [27], which have an unclear etiology and are associated with numerous oncogenes, such as circRAB3IP, RNA00511 [28,29]. It has some tried-and-true and cutting-edge diagnostic methods [30], but their treatment and postoperative functional reconstruction are very challenging. Previously, the standard orthopedic methods for managing malignant bone tumors around the acetabulum were amputation or tumor resection, and prosthesis replacement is widely used in the treatment of the reconstruction of large bone defects around the acetabulum [31]. Other methods, such as allogeneic hemi-socket replacement, proximal femoral eversion implantation, and inactivation of the tumor bone for implantation, have been used to treat primary tumors [32], Innovative therapies, such as fungal-derived materials [33,34], calycosin-exerting potential anti-OS actions [35], have been created in recent years. Nevertheless, there has been a longstanding debate on the best treatment for this condition due to the high rate of postoperative complications. Therefore, the clinical effectiveness of 3D printing technology is of great interest.

3D-printed navigation templates can sigificantly reduce the range of tumor resection and reduce trauma on patients. To compare the accuracy of patient-specific instrumentation (PSI) with the standard manual technique in pelvic tumor resections, Sallent et al. [36] experimented with five female cadaveric pelvises from the Anatomy laboratory. The left pelvis was subjected to PSI osteotomy and the right to the standard manual technique. As a result, PSI improved the accuracy of pelvic tumor resection. Additionally, Heunis et al. [37]used a 3D navigational template to maximize margin resection of the tumor in a patient with osteosarcoma. Eventually, it was demonstrated that it preserved hip stability and critical neurovascular structures.

**Figure 2.**
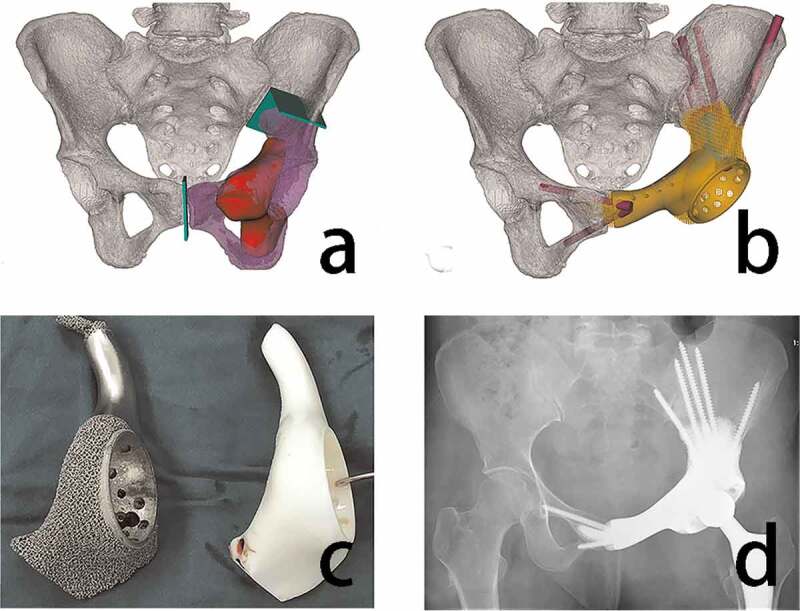
Design, production, and application of 3D printed prosthesis after hip tumor resection (a) osteotomy (green) was simulated on the pelvis model (white), the excised specimen was purple and the tumor was red. (b) the prosthesis was designed based on simulated surgical reconstruction of bone defects, the sacroiliac joint and a part of the pubis were preserved. (c) the endoprosthesis model and prosthesis are exhibited (d) the postoperative plain radiographs showed an accurate reconstruction using a 3D-printed prosthesis [41]

The 3D-printed customized, personalized prostheses conform to the patient’s anatomy, especially in specific areas without a modular prosthesis, maximizing restoration of the acetabulum’s anatomy, and reconstruction of bone defects caused by tumor resection, which restores hip function and reduces the incidence of complications [38,39]. In a recent study, Liang et al. [40]used 3D-printed pelvic prostheses to reconstruct bone defects in 35 patients who had undergone pelvic tumor resection and found that the application of 3D-printing endoprostheses can facilitate precise matching and osseointegration between implants and the host bone. Similarly, Wang et al. [41]used a 3D-printed integrated prosthesis to treat acetabular malignancy, which resulted in improved hip function and reduced postoperative pain, respectively, indicating its significant advantages (Figure 2). Zhu has observed great results when using bespoke 3D printed prostheses for hip environment repair in children with periprosthetic Ewing’s sarcoma excision [42].

## Acetabular fracture

The acetabulum, an essential component of the hip joint, is profoundly concave and hemispherical, consisting of an anterior and posterior column that intersects and arc at 60° [43], and is susceptible to injury because of the high load and mobility of the hip joint. The Judet-Letournel classification divides acetabular fractures into five basic fracture types and five concomitant fracture types, which are used to diagnose acetabular fractures [44]. Afterward, reconstruction of the articular surface and restoration of the anatomy to restore the biomechanical properties of the pelvis and acetabulum are the primary treatments for acetabular fractures [45].

There are usually two types of 3D printed acetabular fracture models: one with an ipsilateral fracture and the other with a complete contralateral mirrored acetabulum. In a retrospective analysis, Yu et al. [46]conducted a comparative study of 146 elderly patients with acetabular fractures, and initial findings showed that the 3D printing mirror model technique was more accurate and had better clinical efficacy.

**Figure 3.**
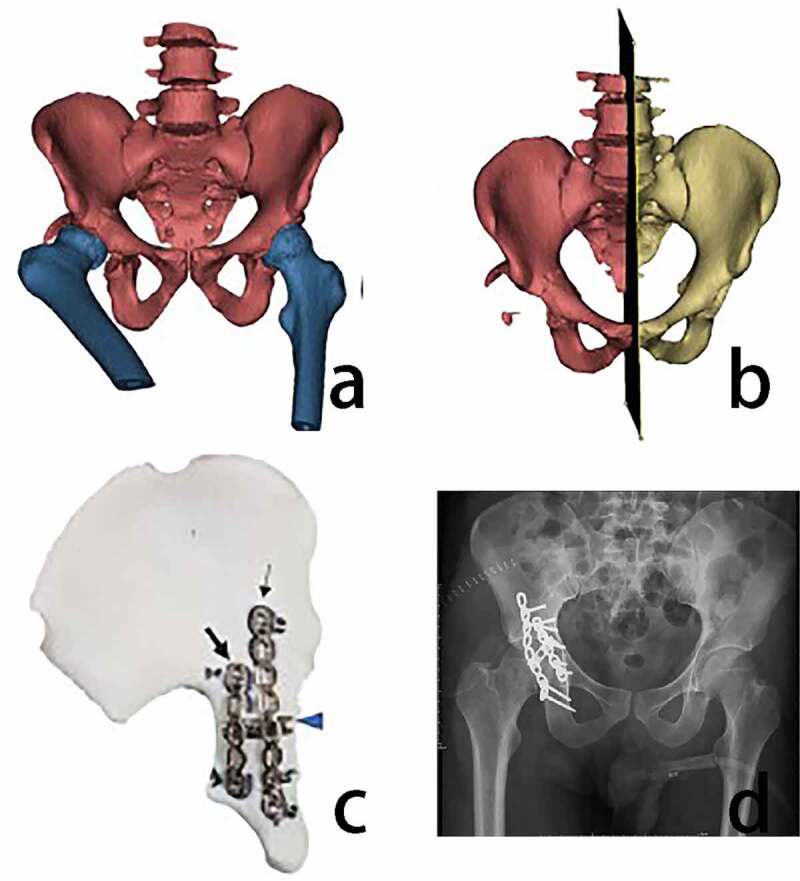
Application of 3D printing technology in the treatment of acetabular fractures (a) 3D image reconstruction of the pelvis and femur of the patient. (b) mirrored reduction of the fractured hemipelvis (c) 3D printed mirror model of the hemipelvis that was used to design pre-contoured plates for internal fixation. (d) follow-up postoperative X-ray [51]

Traditionally, open reduction and internal fixation(ORIF) is considered the ‘gold standard’ for the traditional treatment of unstable acetabular fractures [47]. Still, the acetabulum is deep and difficult to expose, and the surgical incision is long, which requires the protection of vital nerves and blood vessels. In recent years, 3D-printed models have been increasingly used to better understand the anatomy of acetabular fractures [48]. Through clinical studies, many scholars have found that pre-contouring plates with 3D printed models can significantly reduce the time of acetabular fracture surgery and complete fracture fixation faster [49–51](Figure 3). Wan’s treatment of complex acetabular fractures through computerized virtual repositioning combined with 3D printing allows for reduced operative time and postoperative complications compared to conventional surgery, importantly, without lowering the fracture repositioning quality or hip function in patients [43].

**Figure 4.**
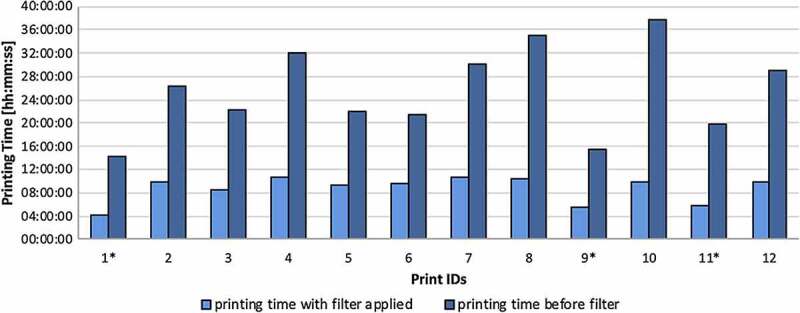
Print times for 12 patients with and without the surface filtering method [53]

The use of navigational templates for the treatment of fractures has also been reported in recent years. Lalit et al. [52] used 3D-printed guides designed for bone screw implantation to treat intra-pelvic fractures, and the data indicated that using a 3D-printed plate significantly reduced the operative time. The limitations of 3D printing also highlight outstanding issues that the printing time is too long, and the surgeon needs to plan ahead when the operation date is confirmed. Weidert et al. [53] created 3D mesh models from CT data of fracture patients using a newly introduced surface-filtering method. They printed the models using PLA materials, resulting in a 65% reduction in the average print time compared to the non-surface filtering method. The plate was flexed preoperatively according to the model, sterilized, and used for surgery. Thus, the Harris hip score improved at follow-up 12 months after surgery (Figure 4).

## Periprosthetic hip joint infection

Periprosthetic joint infection (PJI) is one of the most severe complications of THA. Correct identification of pathogens and timely diagnosis are key to effective PJI treatment [54]. The mortality rate of patients with artificial joint infections is increasing due to older age, poor primary physical conditions, and increased antibiotic resistance to bacteria [55]. When infection occurs, if conventional symptomatic treatment cannot be controlled, surgery should be performed as soon as possible to simultaneously restore the function of the patient’s hip joint and reduce the patient’s pain, which not only creates a burden on families but also seriously affects their quality of life and mental health.

Two-stage reimplantation is the most successful method for treating periprosthetic infections. The first stage involves removing the implant and using a cement gasket containing antibiotics, compounded by intravenous and oral targeting of antibiotics recommended by the doctor for 6–12 weeks. The second phase involves the reimplantation of a new permanent prosthesis [56]. Kim et al. [57]found that 3D-printed liners made of polylactic acid (PLA) performed better than the current bone cement poly(methyl methacrylate) (PMMA) in the treatment of peripheral joint infections, which have superior mechanical properties to PMMA and can elute antibiotics in a controlled manner. This reflects the advantages of RP in prosthetic infections.

**Figure 5.**
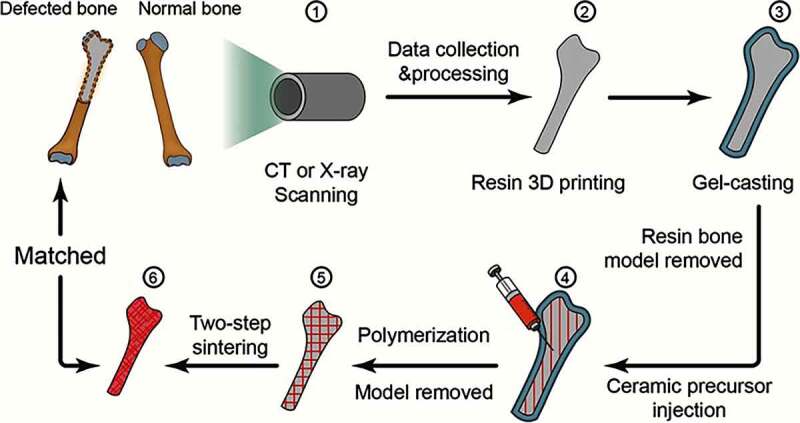
The process of 3D printing technology combined with the gel casting method to manufacture ceramic hip prosthesis [58]

Despite 2-stage revisions, reoperation after infection control in the artificial hip joint prosthesis is the gold standard for the treatment of infection. However, the second operation may result in immense harm to patients. Prosthesis with a better antibacterial effect can decrease the time of operation and joint function recovery in patients by reducing the trauma suffered by the patient. Yang et al. [58]recently combined 3D printing with antimicrobial nano-modification technology to obtain zirconia ceramic implant materials with a precise 3D structure and long-term wear resistance. The prepared hip prosthesis precisely matched the affected part with good biocompatibility and sterilization (Figure 5). In addition, Karaji et al. [59] used electrophoretic deposition of a silk fibroin protein solution consisting of calcium phosphate and vancomycin as a coating on a porous titanium surface made of additives, which exhibited good antimicrobial properties and contributed to bone differentiation. It is believed that the combination of 3D printing technology and antibacterial technology will play an essential role in the treatment of prosthetic infections in the future.

## Conclusions and future perspectives

This review highlights the recent development of 3D printing technology for peripheral hip diseases therapy. Clinical applications of it in orthopedic departments mainly include 3D printing of a bone model, custom prostheses, and navigation templates. With the benefits of 3D printing technology, the operative successful rate of peripheral hip diseases has great potential to increase. It mainly reduces operative time, intraoperative blood loss, and improves joint function.

The widespread application of 3D printing technology in hip diseases provides more surgical information and options, although 3D printing technology still faces many challenges. First, 3D printing technology is costly and has low production efficiency and a long-time cycle. Therefore, it could play a minimal role in emergency treatment. Furthermore, materials with non-toxicity and biomechanical properties, especially high-strength mechanical properties, are required to manufacture prostheses. However, research on these materials is still in the laboratory stage. This will become one of the main directions for the future development of 3D printing technology. In addition, the technology is still not available for printing other articular tissues such as muscle ligaments. There were significant differences between real surgery and simulated surgery due to the lack of surrounding tissues during the simulated surgery. It is prone to unexpected problems during surgery, depending on operator skill. Additionally, although the technology has large sample sizes in clinical applications and the short-term follow-up effect is positive, the long-term follow-up sample sizes are small, so a large amount of long-term follow-up data is crucial to the future evaluation and development of 3D printing technology. Also, a systematic analysis of the study can be conducted in the future, based on a large amount of data.

Although there are some deficiencies in 3D printing technology, 3D printing has proven the capacity to construct structures with varying material composition, structure, and characteristics, providing considerably enhanced performance and usefulness over traditional production processes, which is an indispensible benefit [60]. With the development of bionic materials with biomechanical properties, biocompatibility, and the perfection of 3D printing technology, coupled with the implementation of appropriate national regulatory requirements and the implementation of necessary validation and quality assurance steps, we can look forward to 3D printing technology that will be widely used in the field of joint surgery, providing a new path for the treatment of numerous hip diseases.

## RESEARCH HIGHLIGHTS

1. This article focuses on the application of 3D printing in peripheral hip diseases.
2. The diseases include DDH, acetabular fracture, periacetabular tumors, PJI
3. The types of applications for 3D printing are bone models, navigation templates, PSI.
4. It reduces operative time, intraoperative blood loss, incidence of postoperative complications.
5. What’s more, it improves joint function while reducing surgical damage.
